# Caustic Ingestion With Potassium Thiocyanate

**DOI:** 10.14309/crj.0000000000002101

**Published:** 2026-04-24

**Authors:** Shivani Patel, Ankoor Patel, Anish V. Patel, Courtney Smalley, Steven Brant

**Affiliations:** 1 Division of Gastroenterology and Hepatology, Rutgers Robert Wood Johnson Medical School, New Brunswick, NJ

**Keywords:** caustic ingestion, caustic mucosal injury, potassium thiocyanate, esophagitis, endoscopy

## Abstract

Caustic ingestions are a common cause of injuries to the upper gastrointestinal (GI) tract, most notably the esophagus and stomach. Severity of injury is affected by the nature of the substance, the amount or concentration of the substance, and the duration of direct contact with the mucosa. Typically, cases of caustic ingestion occur more frequently in pediatric patients and in those with psychiatric comorbidities and/or those attempting suicide. Currently, there are no cases in which potassium thiocyanate ingestion resulting in GI sequelae has been reported. We present the first case of caustic mucosal injury to the upper GI tract after potassium thiocyanate ingestion.

## INTRODUCTION

Caustic ingestions are a common cause of injuries to the upper gastrointestinal (GI) tract. Severity of injury is affected by the nature of the substance (alkali or acidic), physical form of the agent, amount or concentration of the substance, and duration of direct contact with the mucosa. Ingestion of toxic substances can lead to GI bleeding, perforation, fistulization, strictures, and can increase the risk of the development of malignancy. To date, no cases of potassium thiocyanate ingestion leading to GI complications have been reported. We present the first case of caustic mucosal injury to the upper GI tract after potassium thiocyanate ingestion.

## CASE REPORT

**Figure 1.**
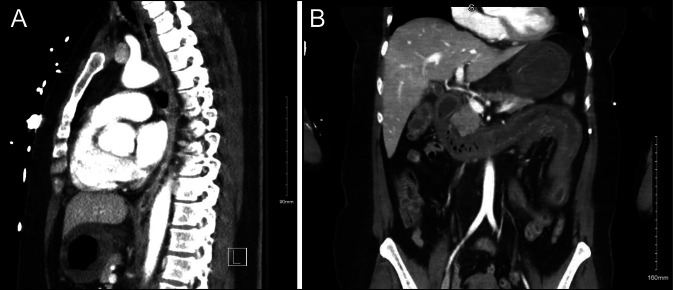
(A) CT scan showing esophageal and gastric wall thickening (sagittal plane). (B) CT scan showing gastric wall and small bowel thickening (coronal plane). CT, computed tomography.

A 65-year-old woman with a history of depression presented after ingestion of an unknown amount of potassium thiocyanate in a presumed suicide attempt 2 hours before arrival. She had multiple episodes of hematemesis and rectal bleeding. On physical examination, the patient was lethargic, with hyperactive bowel sounds, and evidence of dried blood around her mouth, and dark red blood on rectal exam. Bloodwork was concerning for leukocytosis, anemia, hyperkalemia, and worsening high anion gap metabolic acidosis (in the setting of lactic acidosis). Other notable blood work included blood cyanide level 1.130 mg/L. X-rays were negative for pneumomediastinum or pneumoperitoneum. Computed tomography findings demonstrated mild esophageal wall thickening and fluid within the esophagus, severe diffuse wall thickening of the stomach, duodenum, and most of the jejunum with fat stranding and edema (Figure 1).

The case was discussed with poison control, who recommended resuscitation with intravenous fluids and close monitoring of bloodwork, including lactate. Hydroxocobalamin was not advised, given that the patient was hemodynamically stable and a substantially elevated lactate would be expected in cyanide poisoning.^1^ In the setting of encephalopathy, severe metabolic acidosis with hyperkalemia, the patient was intubated, initiated on dialysis, and transferred to the intensive care unit (ICU). It was believed that dialysis would allow for clearance of thiocyanate, which is renally excreted.

**Figure 2.**
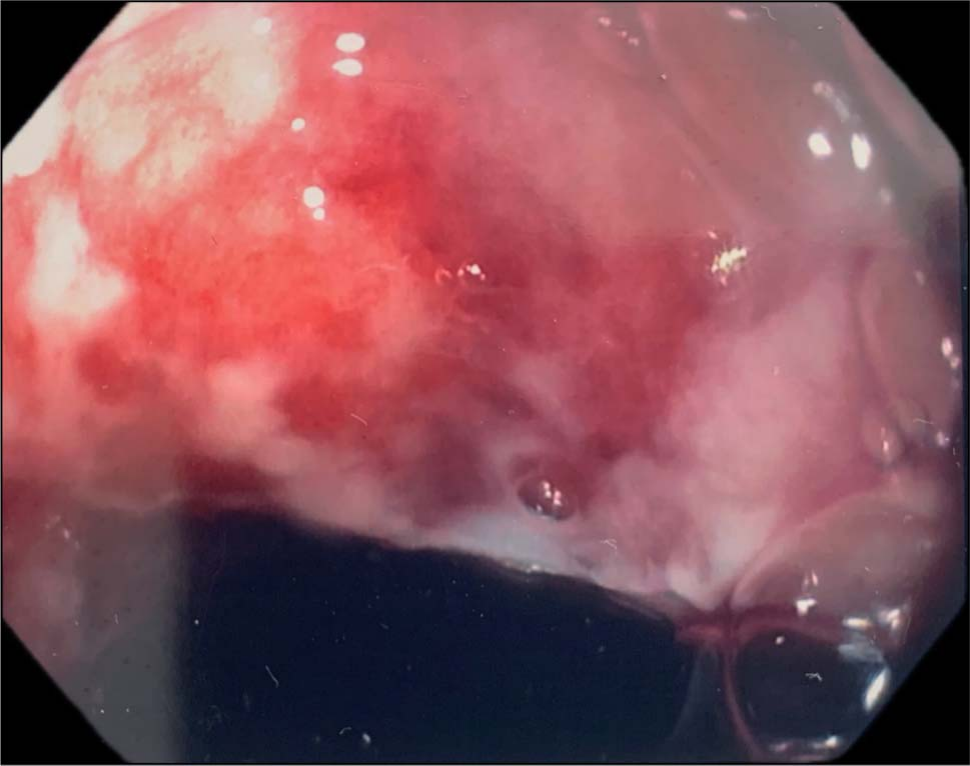
Evidence of superficial ulceration, erosions, friability, and blistering in the distal esophagus (3 cm from the GEJ), classified as Zargar Grade 2a.

**Figure 3.**
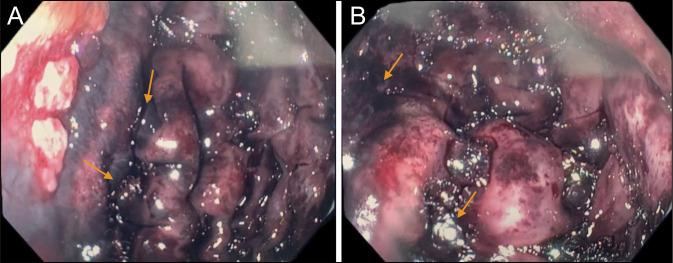
(A) Evidence of scattered areas of necrosis as evidenced by black discoloration along the greater curvature of the stomach, classified as Zargar Grade 3a. (B) Evidence of scattered areas of necrosis as evidenced by black discoloration along the greater curvature of the stomach, classified as Zargar Grade 3a.

Gastroenterology was consulted for an urgent esophagogastroduodenoscopy (EGD) to assess the severity of injury after caustic ingestion. The patient was started on high dose intravenous proton pump inhibitor twice daily on arrival to the emergency department. EGD showed Zargar Caustic Ingestion Injury Grade 1 caustic esophagitis in proximal esophagus, Zargar Caustic Ingestion Injury Grade 2a caustic esophagitis at the distal 3 cm of the esophagus (Figure 2), and Zargar Caustic Ingestion Injury Grade 3a gastritis with diffuse severe mucosal changes with areas of black discoloration and ulceration along entire stomach from both greater and lesser curvature to antrum/prepyloric region (Figure 3). The endoscope was not advanced to the duodenum because of significant concern for perforation, given the extensive necrosis of the stomach. Because of concern for extensive and transmural necrosis of the stomach, the patient underwent immediate exploratory laparotomy revealing a thickened but nonthreatened stomach with an ulcer visualized at the gastroesophageal junction (GEJ) on the posterior aspect of the stomach, 1 cm of the D2 segment of the duodenum appeared threatened but not ischemic or necrotic, and thickening and scattered petechiae of the first 60 cm of the jejunum but did not show evidence of overt necrosis. The colon appeared to be healthy. The abdomen was left open, and the patient returned to the operating room (OR) after 48 hours for abdominal washout and closure. At the time, no further progression of ischemia or necrosis of bowel was noted. The patient's hospital course had been complicated by multiple infections, including ventilator-associated *Enterobacter* and methicillin-susceptible *Staphylococcus aureus* (MSSA) pneumonia and *Escherichia coli* urinary tract infection (UTI), which were treated and resolved. On day 18 of admission, the patient underwent an esophagram, which showed no esophageal leakage and mild delayed contrast transit to the stomach and mild distal esophagitis, gastritis, and duodenitis. On day 19, the nasogastric (NG) tube was removed, and the patient tolerated oral feedings. The patient was subsequently discharged to an outpatient partial hospitalization program on day 24.

## DISCUSSION

Potassium thiocyanate is not a common household chemical and is used in various applications, including as an analytical reagent, for the synthesis of pharmaceuticals and pesticides, as a corrosion inhibitor, and in metallurgy for metal extraction. Upon contact with water, potassium thiocyanate dissolves, releasing the thiocyanate ion (SCN-), which slightly increases hydroxide ion (OH-) concentration, resulting in a pH slightly above 7 and making it a weak base.^2^ Thus far, there have been no reports of ingestion of laboratory-grade potassium thiocyanate. Potassium thiocyanate was used in the early 1900s to treat hypertension. It was known to have side effects of nausea and GI irritation at very low doses but had been linked to neurotoxicity, seizures, and death.^3^ Consumption of 80 mg/kg can induce hallucinations, convulsions, and muscle weakness. At higher doses of 428 mg/kg, side effects also include psychosis and gastritis with ulcerations.^4^

Ingestion of caustic substances in adults is typically intentional, as opposed to children, in whom ingestion is usually accidental, and is often associated with underlying psychiatric illness with an intent to self-harm.^5^ The severity of injury is affected by the properties and volume of the substance. Alkali-induced injury results in a penetrating injury or liquefactive necrosis, as the substance diffuses through the tissue until tissue fluids buffer the alkali. Typically, this results in extensive transmural injury and can cause perforation, mediastinitis, and death. Acid-induced injury typically occurs upon contact and more commonly results in upper airway injuries. Acids induce pylorospasm and chemical stagnation, resulting in injury, particularly in the antrum.^6^ Although food acts as a buffer, acids produce superficial coagulation necrosis and eventually lead to eschar formation.

Ingestion of caustic substances can lead to severe injury of the GI tract, most notably the esophagus, stomach, and proximal small intestine, leading to emergent endoscopic evaluation and surgery. Long-term GI complications after toxic ingestion are predominantly esophagogastric strictures contributing to dysphagia, malnutrition, and the need for serial dilations and/or surgical intervention. Other complications include fistulas as well as an increased risk of development of esophageal carcinoma (predominantly squamous cell cancer) arising in injured segments decades after injury. Guidance of follow-up endoscopy is typically dictated by the extent and severity of injury (using Zargar Classification) and clinical signs of complications (eg, dysphagia, nausea, food intolerance, etc.).^6^ Although there is no universal consensus on the timing of endoscopy, it is typically performed 3–6 weeks after ingestion. Studies suggest initiating surveillance for esophageal cancer approximately 10–20 years after the event, with upper endoscopy every 2–3 years, especially in high-risk patients, which include those with high-grade mucosal injury (Zargar IIb or III) or who have developed strictures.^7^ We report the first case of corrosive esophagitis and gastritis diagnosed via early endoscopy after ingestion of laboratory-grade potassium thiocyanate. Our case highlights the utility of early endoscopic evaluation in predicting immediate and long-term complications and guiding appropriate monitoring and therapy. Endoscopic evaluation and scoring of the severity of injury will help guide future surveillance for late complications after caustic injury, including risk of developing strictures or carcinoma.

## DISCLOSURES

Author contributions: All authors worked in all 4 aspects of authorship as per ICMJE guidelines, including drafting, reviewing, investigating, and conceptualization. S. Brant is the article guarantor.

Financial disclosure: None to report.

Informed consent was obtained for this case report.

## ABBREVIATIONS:

EGD: esophagogastroduodenoscopy
GEJ: gastroesphageal junction
GI: gastrointestinal
ICU: Intensive Care Unit
MSSA: methicillin-susceptible Staphylococcus aureus
NG: nasogastric
OR: operating room
UTI: urinary tract infection
